# Mate-guarding constrains feeding activity but not energetic status of wild male long-tailed macaques (*Macaca fascicularis*)

**DOI:** 10.1007/s00265-013-1673-8

**Published:** 2014-01-11

**Authors:** Cédric Girard-Buttoz, Michael Heistermann, Erdiansyah Rahmi, Anna Marzec, Muhammad Agil, Panji Ahmad Fauzan, Antje Engelhardt

**Affiliations:** 1 Jr. Research Group Primate Sexual Selection, Reproductive Biology Unit, German Primate Centre, Kellnerweg 4, 37077 Göttingen, Germany; 2 Courant Research Centre Evolution of Social Behaviour, Georg-August University, Göttingen, Kellnerweg 6, Germany; 3 Endocrinology Laboratory, German Primate Centre, Kellnerweg 4, 37077 Göttingen, Germany; 4 Faculty of Veterinary Medicine, Syiah Kuala University, Banda Aceh, Indonesia; 5 Faculty of Veterinary Medicine, Bogor Agricultural University, Bogor, Indonesia

**Keywords:** Primates, Sexual selection, Mating costs, Field endocrinology, Feeding behaviour, Urinary C-peptide

## Abstract

Mate-guarding is an important determinant of male reproductive success in a number of species. Little is known however about the constraints of this behaviour, e.g. the associated energetic costs. We investigated these costs in long-tailed macaques where alpha males mate guard females to a lesser extent than predicted by the priority of access model. The study was carried out during two mating periods on three wild groups living in the Gunung Leuser National Park, Indonesia. We combined behavioural observations on males’ locomotion and feeding activity, GPS records of distance travelled and non-invasive measurements of urinary C-peptide (UCP), a physiological indicator of male energetic status. Mate-guarding led to a decrease in feeding time and fruit consumption suggesting a reduced intake of energy. At the same time, vertical locomotion was reduced, which potentially saved energy. These findings, together with the fact that we did not find an effect of mate-guarding on UCP levels, suggest that energy intake and expenditure was balanced during mate-guarding in our study males. Mate-guarding thus seems to not be energetically costly under all circumstances. Given that in strictly seasonal rhesus macaques, high-ranking males lose physical condition over the mating period, we hypothesise that the energetic costs of mate-guarding vary inter-specifically depending on the degree of seasonality and that males of non-strictly seasonal species might be better adapted to maintain balanced energetic condition year-round. Finally, our results illustrate the importance of combining behavioural assessments of both energy intake and expenditure with physiological measures when investigating energetic costs of behavioural strategies.

## Introduction

In a broad range of taxa (e.g. insects, Alcock 1994; reptiles, Censky 1995; Ancona et al. 2010; crustaceans, Sparkes et al. 1996; birds, Komdeur 2001; Low 2006; mammals, Alberts et al. 1996; Matsubara 2003; Willis and Dill 2007; Schubert et al. 2009), males have evolved mate-guarding behaviour to exclude rivals from mating with the guarded female. In this way, males can limit the extent of sperm competition (i.e. sperm from different males competing to fertilise one particular ovum, Birkhead and Møller 1998) and increase their chances to fertilise the female. Mate-guarding can take different forms, such as: (1) prolonged copulation beyond the time required for fertilisation (Carroll 1991), (2) maintenance of permanent physical contact with the female (female grasping) or continuous monitoring of the female after mating to prevent the female from mating with other males (Alberts et al. 1996; Sparkes et al. 1996) and (3) formation of mating plugs sealing the access to the female genital tract (Alcock 1994). In this paper, we focused on the second form of mate-guarding and define it as a ‘close, persistent following of a female by a male that involves exclusion of other males from access to the female’ (cf. Alberts et al. 1996).

While mate-guarding is usually highly beneficial to males, as it significantly increases mating and/or reproductive success (Censky 1995; del Castillo 2003; Setchell and Kappeler 2003; Engelhardt et al. 2006), maintaining a permanent physical contact with the female or monitoring female activity after mating has proven to be costly in several taxa. Mate-guarding can affect males’ thermoregulation (Singer 1987; Saeki et al. 2005), feeding behaviour (Censky 1995; Alberts et al. 1996; Komdeur 2001; Ancona et al. 2010) and fat reserves (Komdeur 2001; Plaistow et al. 2003; Schubert et al. 2009).

In primates, mate-guarding is a common reproductive strategy in species living in multi-male multi-female groups (reviewed in Manson 1997). However, the intensity of mate-guarding, i.e. its duration and the diligence with which males engage into this behaviour, varies greatly across species (Manson 1997). This variation is most likely due to interspecific differences in costs and benefits of mate-guarding. It has been suggested that the cost–benefit ratio of primate mate-guarding depends on variation in demographic and ecological factors as well as detectability of the female fertile phase, which in turn influence female monopolisability and the degree of male–male contest competition (Dubuc et al., unpublished data). Interestingly, the degree of male–male competition and of female reproductive synchrony appear to be positively correlated to the degree of reproductive skew (Ostner et al. 2008; Gogarten and Koenig 2013) suggesting that both parameters, male reproductive skew and the intensity of mate-guarding, are linked to each other in primates. Understanding the constraints of male mate-guarding abilities may thus also foster our understanding of what determines male reproductive skew in primates.

A model commonly used to explain the variation in male reproductive skew in primates is the Priority of Access model (PoA model, Altmann 1962). According to this model, the limiting factor to male mating and reproductive success is female cycle synchrony. As a male is only able to mate guard one female at a time, the model predicts that male reproductive skew depends on the number of females cycling at the same time. Whereas data support the PoA model in some primate species (savannah baboons (*Papio cynocephalus*), Altmann et al. 1996; Alberts et al. 2003; mandrills (*Mandrillus sphinx*), Setchell et al. 2005; and chimpanzees (*Pan troglodytes*), Boesch et al. 2006), in others, mate-guarding behaviour is less intense and reproductive skew is lower than predicted by the model (e.g. long-tailed macaques (*Macaca fascicularis*), Engelhardt et al. 2006; rhesus macaques (*Macaca mulatta*), Dubuc et al. 2011; and Barbary macaques (*Macaca sylvanus*), Young et al. 2013a). This indicates that other variables than female cycle synchrony may explain the degree of male reproductive skew. The most commonly proposed variables are the energetic costs of mate-guarding and the ability of males to detect the period when the female is fertile (Alberts et al. 1996; Engelhardt et al. 2006; Dubuc et al., unpublished data). However, whereas the ability of males to discern the female fertile phase has been investigated in a number of primate species (e.g. Heistermann et al. 2001; Deschner et al. 2004; Engelhardt et al. 2004; Dubuc et al. 2012; Higham et al. 2012; Young et al. 2013b), the specific energetic costs of mate-guarding remain largely unclear.

A number of studies on primates have tried to accurately quantify the impact of mate-guarding on key components of male energy intake (feeding duration and ingestion rate) and expenditure (distance travelled) but the results from these studies are inconclusive. Whereas, in a number of studies, mate-guarding led to a reduction in male-feeding time (Packer 1979; Rasmussen 1985; Alberts et al. 1996; Matsubara 2003), evidence is equivocal in chacma baboons (*P. hamadryas ursinus*: Weingrill et al. 2003), and completely absent in olive baboons (*Papio anubis*, Bercovitch 1983), Assamese macaques (*M. assamensis*, Schülke et al. 2013) and moustached tamarins (*Saguinus mystax*, Huck et al. 2004 *)*. Furthermore, only one study so far investigated energy expenditure in relation to mate-guarding (Alberts et al. 1996) finding that male baboons travelled less distance when mate-guarding females than when not. Finally, only one study investigated potential costs to mate-guarding other than energetic ones, such as elevation of physiological stress levels. In chacma baboons, males had higher faecal glucocorticoid levels (a marker of physiological stress) when mate-guarding females than when not (Bergman et al. 2005).

None of these aforementioned studies controlled for important factors known to influence activity patterns and feeding strategies of primates such as variation in food availability (Masi et al. 2009; Chaves et al. 2011). More importantly, the lack of physiological measures in these studies prevents clear conclusions about male energetic status since behavioural observations may be rather inaccurate in this regard (Chivers 1998).

A potentially useful tool to assess energetic condition more accurately is the measurement of urinary C-peptide (UCP), a small polypeptide produced in an equimolar ratio when the body converts proinsulin into insulin (Rubenstein et al. 1969). UCP has been validated as a reliable non-invasive measure of energetic status in non-human primates with higher UCP levels reflecting better body condition and positive energy balance (Sherry and Ellison 2007; Deschner et al. 2008; Emery Thompson and Knott 2008; Girard-Buttoz et al. 2011). Its use to investigate the energetics of reproductive strategies in male primates is so far restricted to one single study on free-ranging male rhesus macaques. In these males, UCP levels decreased significantly during the mating season, and this more in high-ranking than in low-ranking males (Higham et al. 2011a). In addition to quantifying the effect of dominance rank on male energetic status, the relationship between UCP levels and male restlessness, a behaviour defined as the rate of changes between different activities, was also investigated. It turned out that restlessness was negatively associated with UCP measures in these males. Restlessness has rarely been studied in animals, but these results indicate that it is possibly an important, so far overlooked, source of energy expenditure. Individual variation in restlessness thus needs to be included into the analysis of energetic costs.

Given the lack of fully conclusive studies on the costs of mate-guarding in primates, the aim of our study was to quantify the energetic costs of this male reproductive strategy with a more comprehensive approach using the long-tailed macaque as a model species. Long-tailed macaques are organised in multi-male groups and are non-strictly seasonal breeders (van Schaik and van Noordwijk 1985). Although female long-tailed macaques can conceive year-round, periods with increased female sexual receptivity (mating period) and birth peaks frequently occur, the timing of which seems to depend on fruit availability (van Schaik and van Noordwijk 1985). The species is therefore classified as capital breeder on the three grade scale of reproductive seasonality in primates by Brockman and van Schaik (2005). In long-tailed macaques, males seem able to discern a female’s fertile phase (Engelhardt et al. 2004) and alpha and beta males are the only males to mate-guard females intensively (de Ruiter et al. 1994; Engelhardt et al. 2006). Mate-guarding intensity translates into male reproductive success, which is highly skewed towards high-ranking males (de Ruiter et al. 1994; Engelhardt et al. 2006). However, alpha males do not mate guard all potentially fertile females even when fertile phases do not overlap (Engelhardt et al. 2006). This finding strongly suggests that alpha male ability to monopolise all females one after the other may be limited because of some costs associated with mate-guarding behaviours among which energetic costs might be the major limiting factor.

In order to better understand the energetic dynamics of mate-guarding in male long-tailed macaques, we quantified potential energetic costs in various ways. We investigated the effects of mate-guarding on some key components of energy intake and expenditure: (1) feeding time, (2) diet, (3) travelling and climbing distance and (4) restlessness. Furthermore, we used UCP measures to investigate whether mate-guarding intensity affects male energetic status overall. Potential confounding effects of environmental factors (fruit availability and rainfall) were controlled for in all analyses. This study will provide useful insight into male energetic management during reproductive competition.

## Methods

### Animals and study site

The study was carried out from January 2010 to April 2011 on three groups of long-tailed macaques living around the Ketambe Research Station (3°41′ N, 97°39′ E), Gunung Leuser National Park, North-Sumatra, Indonesia. The study area consists of primary lowland rainforest and has been described by Rijksen (1978) and van Schaik and Mirmanto (1985). The long-tailed macaques in the area have been studied since 1979 (van Schaik and van Noordwijk 1985; de Ruiter et al. 1994; Engelhardt et al. 2004). We focused on three groups: Camp (C), Ketambe Bawa (KB) and Ketambe Atas (KA). All adult individuals were individually known and well habituated to human observers. The total size of a social group varied from 22 to 58 individuals (see Table 1 for group compositions and home range sizes). Between January and April 2010, four males migrated back and forth between the groups KA and KB and associated with the group for periods between a few hours up to 3 weeks before migrating back to their ‘home’ group. The study was conducted completely non-invasively and under the permission of the authorities of Indonesia. We adhered to the Guidelines of the Use of Animals in Research, the legal requirements of Indonesia and the guidelines of the involved institutes.

**Table 1:** Composition and home range of the study groups

Group	Females (*N*)	Males (*N*)	Total (*N*)	Home range size (ha)
Camp	14–15	6–9	54–58	34.3
Ketambe Bawa	9–10	4–8	31–36	20.3
Ketambe Atas	7	4–7	22–25	19.1

### Behavioural data

Behavioural data were collected by CG-B and six experienced Indonesian and international field assistants. The observation periods covered two mating periods. Each mating period was defined as the period between the first mate-guarding day and the last mate-guarding day observed in any of the three groups. From March to July 2010, four observers followed groups C and KB every day and from December 2010 until April 2011 all three groups were generally followed every other day and frequency of observations increased to every day when alpha and/or beta males were observed mate-guarding.

We focused our focal behavioural observations on alpha and beta males of each group because they are the ones to mate-guard females most extensively (Engelhardt et al. 2006). All behavioural data were recorded using a handheld computer Psion Workabout Pro (Teklogix®). Every evening, the identity of the males to observe the next day was determined based on the mate-guarding activity of each male and on whether they were followed or not that day. Males were then followed half or full day depending on the number of observer available and on the number of males to follow. Once the behavioural sampling procedure was decided, full- or half-day focal protocols were completed regardless of whether the focal male was mate-guarding females or not. Accordingly, our protocol does not influence the percentage of time during which a male was observed mate-guarding on a given day. Each day, groups were followed from dawn to dusk.

Dominance ranks between males were determined using the ‘bared-teeth-face’ display, a unidirectional submissive display (van Hooff 1967). All occurrence of bared-teeth-face observed between any male of the group were recorded during the focal protocols. Bared-teeth-face giver and receiver were entered into a sociometric matrix and dominance ranks were compiled with Matman 1.1.4 using the I&SI method (de Vries 1998). The following focal scan data (Altmann 1974) were recorded every minute: activity (resting, being vigilant, feeding (handling and consuming food), drinking, travelling (continuous locomotion during at least 1 min with no foraging activity and no social interactions), aggressing, affiliating (including copulation), grooming, self-grooming), locomotion/position (lying, sitting, standing, walking, running, jumping and climbing), canopy height (six categories: **0** = focal animal on the ground, **1** = 1–5 m; **2** = 5–10 m; **3** = 10–15 m; **4** = 15–20 m; **5** = 20–25 m; **6** = >25 m) and, if the animal was feeding, the food item was recorded (fruit, leaf, flower, arthropod, bark, mushroom or others). Activity states ‘resting’, ‘being vigilant’, ‘feeding’, ‘drinking’, ‘aggression’ and ‘affiliation’ were mutually exclusive. However ‘grooming’, ‘self-grooming’, ‘feeding’ and ‘travelling’ were not (e.g. the animal could be eating and groomed at the same time). When the focal animal was simultaneously engaged in feeding and another activity (e.g. grooming), we categorised the activity as ‘feeding’ because the focus of our study is on energetics. At 1 min intervals, we also recorded whether the male was following a female and, if so, we recorded the identity of the female and the distance between them. In addition, all copulations were recorded.

The 1-min scans during which the male was mate-guarding were determined à posteriori. A male was considered as ‘mate-guarding’ when he followed a female for more than five consecutive minutes and maintained a distance of less than or equal to 10 m between him and the female. If the female moved farther away than 10 m from the male and the male did not follow her for more than 2 min, the mate-guarding activity was considered to have ended. We termed days during which the male mate-guarded females more than 50 % of the observation time ‘extensive mate-guarding days’. A mate-guarding period was defined as a period of one to several consecutive extensive mate-guarding days.

### Travelling distance

Every day, the position of two of the focal males among the three groups was recorded automatically every minute using the tracking function of a GPS (Garmin© GPSMAP 60csx). Due to canopy density, the precision of GPS locations were only ±10 m (the average measurement error of the GPS was calculated by recording the measurement error displayed on the GPS directly in the field during the first week of the study). We extracted GPS location every 15 ± 5 min and calculated the distance between each 15-min location. This approach reduces the average measurement error per hour from 600 (when recording location every minute) to 40 m (when recording location every 15 min), while keeping precise information on the focal individual path length. We used only GPS points for which we had focal behaviour on the activity of the animal at the exact minute at which the GPS point were recorded (i.e. when the observer was directly below the animal). For each day, the average horizontal distance travelled per hour was then calculated as ${\displaystyle \sum_{i=1}^n}\frac{4* Di}{\ n}$ where *D*_*i*_ is the distance between two consecutive locations and n the number of distances recorded that day. *D*_*i*_ is the distance travelled during each 15-min interval. The sum of *D*_*i*_ divided by *n* is the average distance travelled by the animal during 15 min. By multiplying this sum by 4, we obtain an average hourly travelling distance.

In order to calculate the vertical distance travelled, we first used the centre of each height category as an estimate for the height at which the male was at each minute-scan point (e.g. 7.5 m for category 2 or 12.5 m for category 3). Subsequently, we calculated the height difference between each minute-scan-height estimate.

### Fruit availability

In each of the three studied groups, 40 locations, covering the entire home ranges, were randomly selected (120 locations in total over the three territories). At each location, three trees were randomly selected from three different species among the tree species producing fruit eaten by *M. fascicularis* (Ungar 1995). For each tree, the height and the DBH (diameter at breast height) were recorded using a laser range finder and a flexible tape measure with 1 mm gradations (see Table 2). In total, 360 trees from 87 different species were selected (120 trees for each group’s home range). Each tree was surveyed monthly within the last 3 days of every month by an experienced field assistant and fruit abundance was recorded using a logarithmic scale (0, absence; 1, 1–10 items; 2, 11–100; 3, 101–1,000; 4, 1,001–10,000; 5, >10,000, a method commonly used in primatology, Ganzhorn 2003). The average monthly score of fruit abundance in each territory was highly correlated to the percentage of trees fruiting. For the analyses, we therefore used percentage of trees fruiting as an index of fruit availability.

**Table 2:** Species diversity and morphological characteristics of the 360 trees surveyed for assessing fruit availability

Group	Camp	Ketambe Atas	Ketambe Bawa
Number of tree species	55	55	59
Mean tree dbh (cm)	41.3	26.9	36.7
Mean tree height (m)	17.4	14.3	16.7

*dbh* diameter at breast height

### Urine sample collection

Urine samples were generally collected once a week from four males in each group: alpha and beta males and two low-ranking males as ‘controls’ (males which do not mate-guard females extensively). In addition, during mate-guarding periods, we collected urine samples every other day from the mate-guarding male. A large plastic sheet (100*50 cm) was placed beneath urinating animals. Urine was then pipetted from the plastic sheet and/or from the vegetation directly after urination and placed in a 2-ml Eppendorf safe-lock tube. Right after collection, the urine samples were placed on ice in a thermos bottle. At the end of each fieldwork day, the samples were frozen at−20 °C in a freezer kept under constant electricity supply. As such, the samples were never stored on ice for more than 12 consecutive hours, a duration which does not significantly affect UCP concentrations (Higham et al. 2011b). In July 2011, all the samples were transported, on ice, to the hormone laboratory of the Bogor Agricultural University (IPB) and then freeze dried before transportation to the Reproductive Biology Unit of the German Primate Centre for C-peptide analysis. Prior to lyophilisation the volume of urine in each sample was assessed visually to the nearest 0.1 ml.

### C-peptide analysis

As C-peptide levels potentially become unreliable when stored long term (i.e. >8 months, see Higham et al. 2011b), all samples that were stored frozen longer than 8 months before lyophilisation were excluded from analyses. Accordingly, only the samples collected during the second mating period (December 2010 to April 2011) were used. C-peptide concentrations were measured using a commercial C-peptide ELISA Kit from IBL International GmbH, Hamburg, Germany (Art. No. RE 53011), which we recently validated for the use in macaques (Girard-Buttoz et al. 2011). Prior to assay, urine samples were reconstituted into 0.3 (samples with a urine volume prior to lyophilisation, <0.5 ml) or 0.5 ml (volume prior to lyophilisation, >0.5 ml) of distilled water by vortexing the samples for 90 s. The samples were reconstituted in less volume than the original volume of fresh urine (i.e. prior to lyophilisation) in order to concentrate the sample and increase the chances of UCP measures to be above assay sensitivity (i.e. 0.064 ng/ml). Reconstituting the samples in a different volume from the original ones does not affect UCP measures since these measures are indexed to creatinine concentration (see below); 100 μl of the urine was then assayed using the manufacturer provided protocol. Inter-assay coefficients of variation calculated from the measurement of low, middle and high value quality controls ran in each assay were 9.0, 7.0 and 8.4 %. Intra-assay coefficients of variation provided by the manufacturer are 6.5, 6.7 and 5.1 %.

To adjust for differences in urine concentration, C-peptide values were indexed to urinary creatinine measured according to the method described by Bahr et al. (2000). Assay sensitivity was 0.1 mg/ml. Inter-assay coefficients of variation calculated from the measurement of low and high value quality controls ran in each assay were 0.6 and 1.4 %. C-peptide concentrations are presented as ng C-peptide/mg creatinine.

### Statistical analyses

For all analyses, we considered only those days of observation for which at least 1 h of focal data were recorded. The final data set thus comprised 2,088 h of focal observations over 584 days (see Table 3).

**Table 3:** Observation time, mate-guarding period length and diversity of females mate guarded by the study males

Group	Camp		Ketambe Atas		Ketambe Bawa	
Male rank	α	β	α	β	α	Β
Number of mating periods	2	2	1	1	2	2
Focal observation time (hours)	668	455	185	111	388	323
Number of days of observation	147	114	68	48	122	85
Number of urine samples	45	25	27	19	32	22
Number of females mate guarded	5	3	3	2	8	5
Number of MG days	41	4	30	27	49	10
Mean MG period length (days)	3.9	1	4.9	9	3.7	1.5
Range of MG period length (days)	1–18	1–1	1–13	1–33	1–10	1–4
Overall MG time	27.4 %	8.4 %	40.2 %	53.9 %	36.9 %	12.0 %

The overall MG time is the percentage of observation time during which the male was mate-guarding females

*MG* mate-guarding, *MG days* days during which the males were mate-guarding female for more than 50 % of observation time

#### Influence of mate-guarding on male behaviour and energetic status

We considered fruit to be a relevant to energy intake for long-tailed macaques. In fact, in this species, females time their reproduction according to seasonal variation in fruit abundance, illustrating that fruit fulfils important energy requirements for this species (van Schaik and van Noordwijk 1985). We therefore included percentage of fruit in the diet into our analysis. For each day, we calculated the percentage of time spent mate-guarding and feeding, the percentage of fruit in the diet as well as the distance travelled per hour (in metres), the average height difference per minute (in metres) and the rate of changes in locomotion/position (restlessness) as behavioural measures of energy intake and expenditure. In addition, restlessness, another important factor of energy expenditure (see Higham et al. 2011a), was derived by generating a binary matrix of changes in either position or locomotion (locomotion/position was entered in a single column). For each minute of observation, the matrix was filled with a 0 if the locomotion or position was the same as the minute before. Otherwise, it was filled with a 1. For each day, we calculated the rate of changes by dividing the total number of ‘1’s’ by the total number of ‘0’s’ and ‘1’s’. In other words, the possible restlessness score ranged from 0 to 1, with 0 indicating a complete absence of restlessness and 1 indicating maximal restlessness.

We tested whether the percentage of time spent mate-guarding on a given day affected the following parameters in the males: (1) time spent feeding, (2) percentage of fruit in the diet, (3) distance travelled, (4) height climbed, (5) restlessness and (6) energetic status (as assessed by UCP measures). We used a generalised linear mixed model (GLMM, Baayen 2008) for each of the parameters to test whether, on a particular day, this parameter was influenced by the percentage of time spent mate-guarding.

As ecological conditions are likely to affect these parameters, we included fruit availability as a control factor in each model. Fruit availability on a given day was approximated using the fruit availability measured on the closest monthly record. For example, the percentage of tree fruiting recorded on the 31st of January was used as the fruit availability score for all the days between the 16th of January and the 15th of February.

We also included daily rainfall in each model as, because of thermoregulation constraints, this variable can influence primate locomotion and activity budget (Ganas and Robbins 2005; Majolo et al. 2013) and ultimately their energetic status. Therefore, we included the percentage of time spent mate-guarding, fruit availability, their two-way interaction and rainfall as fixed effects and male ID and group as nested random effects. In all the GLMMs, the interaction between fruit availability and percentage of time spent mate-guarding was never significant (for all tests, *P* > 0.05). Therefore, we re-ran all the models without the interaction and all the results presented are for models with no interaction. For the model with UCP levels as response variable we added time of sample collection (to account for possible diurnal variability in our physiological measure), duration of sample storage in the freezer, height climbed (to account for variation in energy expenditure) and feeding time (to account for variation in energy intake) as control variables. All continuous fixed factors in the models were standardised to a standard deviation of 1 and mean of 0. All models were fitted in R 2.15.0 (R Development Core Team 2010) using the function lmer of the R-package lme4 (Bates and Maechler 2010).

The response variable in the different models was likely to show temporal autocorrelation unexplained by the fixed effects included, potentially leading to violation of the assumption of independent residuals. Therefore, we included a temporal autocorrelation term into the model using an approach developed by Roger Mundry (see Fürtbauer et al. 2011).

In each model, we checked that the assumptions of normally distributed and homogeneous residuals were fulfilled by visually inspecting a qqplot and the residuals plotted against fitted values. We checked for model stability using bootstraping by excluding data points one by one from the data and comparing the estimates derived with those obtained for the full model. We checked that the range of the estimates derived were symmetric around the estimate value of the full model, which indicates the absence of influential cases. Variance inflation factors (VIF; Field 2005) were derived using the function VIF of the R-package car (Fox and Weisberg 2010) applied to a standard linear model excluding the random effect. VIF’s which are less than 5 indicate that covariation between the predictors is not strong enough to violate the assumptions of the model (Bowerman and O’Connell 1990; Myers 1990). In all our models, VIF’s were less than 1.4. The other diagnostics also did not indicate obvious violation of the assumption.

For each model, we first determined the significance of the full model (including all fixed effects, the interaction, the autocorrelation term and the random effects) as compared with the corresponding null model (including all the factors except ‘the percentage of time spent mate-guarding’) using a likelihood ratio test (R function anova with argument test set to ‘Chisq’). To achieve a more reliable *P* value, we fitted the models using maximum likelihood (rather than restricted maximum likelihood, Bolker et al. 2009). Only if this likelihood ratio test revealed significance did we consider the significance of the individual predictors. *P* values for the individual effects were based on Markov chain Monte Carlo sampling (Baayen 2008) and derived using the functions pvals.fnc and aovlmer.fnc of the R-package languageR (Baayen 2010).

#### Influence of rank on male energetic status

To test whether males who mate-guarded females extensively during the mating period (alpha and beta males) had a lower energetic status than other males, we ran a GLMM including UCP levels as a response, male rank (two categories: high-ranking for alpha and beta males, and low-ranking for two other males in the same group) as a fixed factor, time of sample collection and sample storage length as control fixed effects and animal ID and group as random factors. In this model, rank was used as a proxy of mate-guarding intensity since we did not record behavioural data on low-ranking males. However, previous studies showed that alpha and beta males are the only ones mate-guarding females intensively (de Ruiter et al. 1994; Engelhardt et al. 2006). The GLMM was calculated and the assumptions checked as described above.

## Results

### Mate-guarding activity

In each of the three groups, the alpha male mate guarded a higher number of females than the beta male (Table 3). Males mate guarded each female on average four consecutive days (range, 1–33; Table 3) and on average 29.8 % (range, 8.4–53.9 %; Table 3) of their time was devoted to this behaviour.

### Mate-guarding and feeding behaviour

**Fig. 1:**
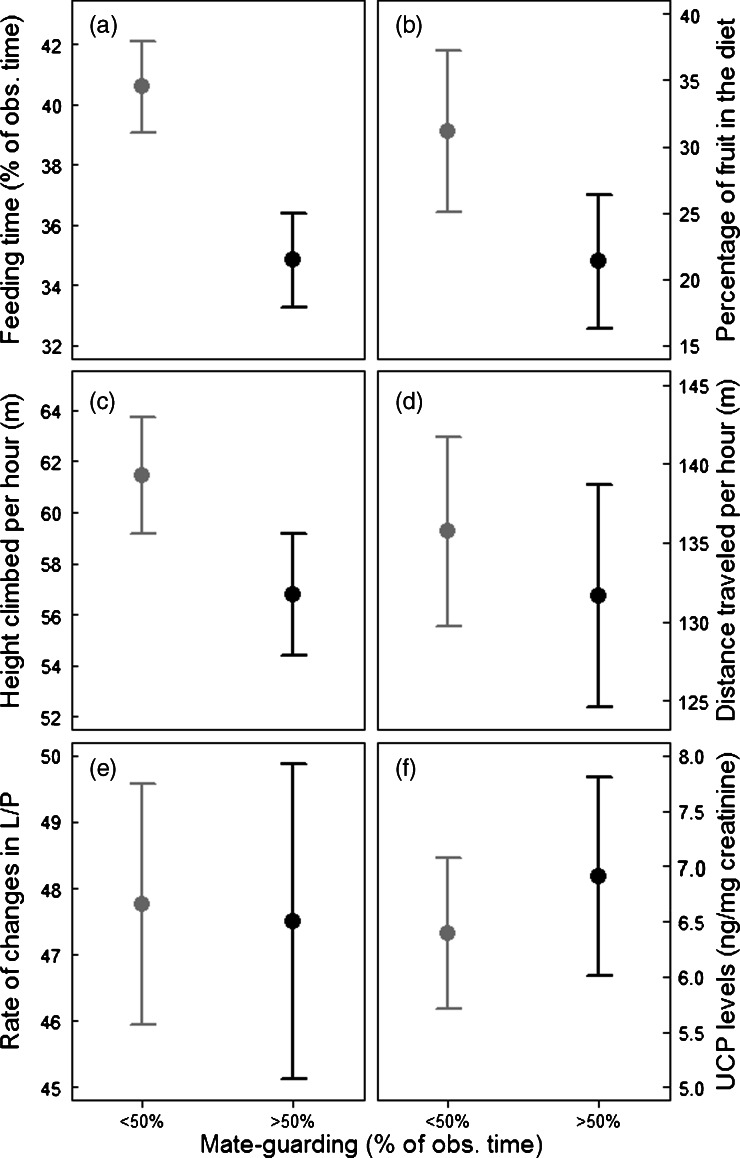
Influence of mate-guarding intensity (<50 % of observation time on the left in *grey* and >50 % of observation time on the right in *black*) on males’ **a** feeding time, **b** percentage of fruits in the diet, **c** height climbed per hour, **d** distance travelled per hour, **e** rate of change in locomotion/position (restlessness) and **f** UCP levels (energetic status). The mean ± SE over all males is depicted for each of the parameters. Please note that these graphs are no substitute for the statistical models presented in Tables 4 and 5

**Fig. 2:**
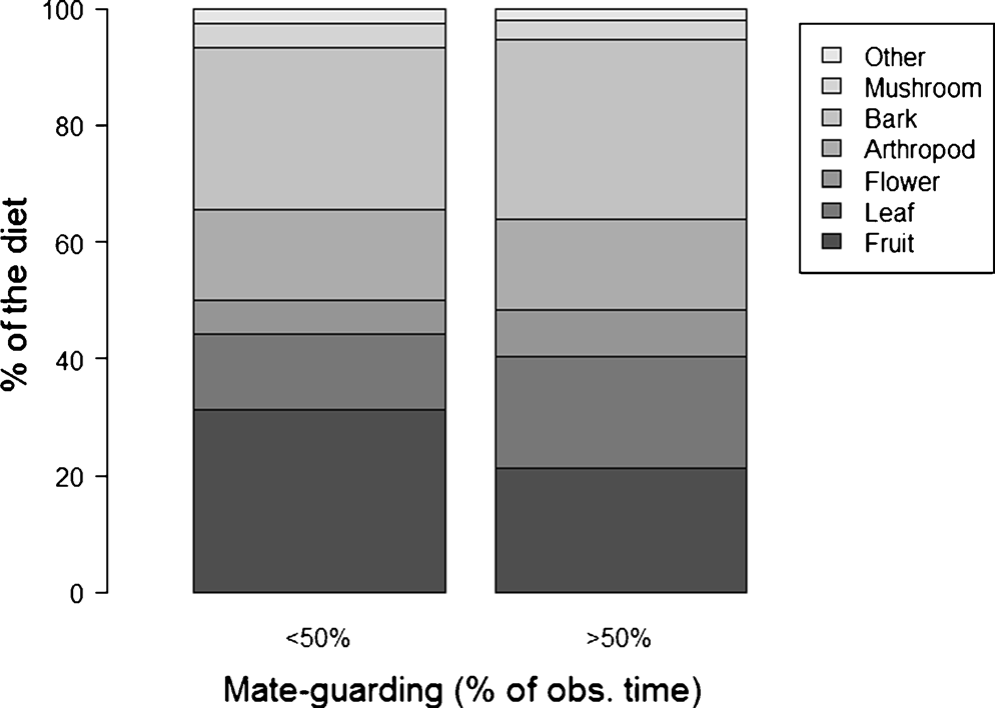
Male diet on the day during which they mate-guarded females less than 50 % (*left bar*) and more than 50 % (*right bar*) of the observation time

The amount of time spent mate-guarding had a strong negative effect on the amount of time males spent feeding (Tables 4 and 5; Fig. 1a). Mate-guarding activity also had a strong effect on the percentage of fruit in the diet (Tables 4 and 5; Fig. 1b). In particular, males fed less on fruits and more on leaves and flowers when mate-guarding females extensively than when not (Fig. 2).

**Table 4:** Results of the Likelihood ratio tests (LRT) run to compare full versus null models of the different GLMMs to test the influence of mate-guarding activity on different behavioural and physiological parameters

Model response	Males (*N*)	Observation days (*N*)	Null vs. full model		
			*df*	*χ* ^*2*^	*P*
% time feeding	6	584	1	14.58	<0.001
% fruit in the diet	6	583	1	8.99	0.003
Height climbed	6	584	1	4.39	0.036
Distance travelled	6	413	1	1.48	0.224
Restlessness	6	580	1	0.89	0.343

**Table 5:** Estimates ± SE, *t*-value and MCMC *P* values of the different GLMMs to test the influence of mate-guarding activity on different behavioural parameters

Model Response		Predictor variables			
		Time spent mate-guarding	Fruit availability	Rainfall	Autocorrelation term
% Time feeding	Estimate ± SE	−2.16±0.56	−1.21±0.56	0.22±0.55	3.33±0.55
	*t* value	−3.84	−2.15	0.42	6.08
	*P*_*MCMC*_	*<0.001*	*0.023*	0.711	*<0.001*
% Fruit in the diet	Estimate ± SE	−2.97±0.96	7.08±1.07	−0.95±0.90	5.57±0.91
	*t* value	−3.11	6.63	−1.05	6.15
	*P*_*MCMC*_	*0.002*	*<0.001*	0.298	*<0.001*
Height climbed	Estimate ± SE	−1.76±0.86	−1.87±0.95	−1.18±0.82	3.38±0.83
	*t* value	−2.05	−1.97	−1.43	4.08
	*P*_*MCMC*_	*0.042*	*0.049*	0.153	*<0.001*
Distance travelled^a^	Estimate ± SE	−2.51±2.19	−2.72±2.40	−2.79±2.02	−3.82±2.12
	*t* value	−1.15	−1.13	−1.38	−1.80
Restlessness^a^	Estimate ± SE	−0.37±0.39	0.12±0.43	−0.07±0.38	0.29±0.37
	*t* value	−0.95	0.28	−0.18	0.79

^a^The MCMC *P values* are not shown for the models in which the full model were not significantly different from the null model (LRT, *p* > 0.05)

### Mate-guarding and energy expenditure

Males travelled on average 1.40–1.96 km/day (i.e. 117–163 m/h over 12 h). The amount of time spent mate-guarding had a statistically significant negative effect on the distance climbed per hour (Tables 4 and 5; Fig. 1c) but did not significantly affect the distance travelled per hour (Table 4; Fig. 1d) or male’s restlessness (Table 4; Fig. 1e).

### Male energetic status

Neither the amount of time spent mate-guarding (Table 6; Fig. 1f) nor male dominance status (Table 6) had a statistically significant influence on UCP levels. In both models, none of the control variables significantly affected the UCP levels. However, height climbed tended to be negatively correlated with UCP levels in the first model (mate-guarding (UCP model); Table 6).

**Table 6:** Results of the Likelihood ratio tests (LRT) run to compare full versus null models, estimates ± SE, *t*-value and *P* values for the two GLMMs run to test 1/the influence of mate-guarding activity UCP levels and 2/the influence of male rank on UCP levels

	1/ UCP – Mate-Guarding Model			2/ UCP – Rank Model		
N. Samples	170			331		
Null vs. full model	*df*	*χ* ^*2*^	*P*	*df*	*χ* ^*2*^	*P*
	1	0.14	0.706	1	0.35	0.552
	Estimate±SE	*t*	*P*_*MCMC*_	Estimate±SE	*t*	*P*_*MCMC*_
MG time	0.04±0.10	0.38	0.703			
Height climbed	−0.21±0.11	−1.84	0.068			
Feeding time	0.03±0.12	0.25	0.767			
Rank				0.14±0.15	0.93	0.355
% Tree fruiting	−0.20±0.19	−1.05	0.293	−0.15±0.10	−1.47	0.142
Time of sample collection	0.06±0.11	0.55	0.582	0.07±0.07	0.92	0.357
Storage length	0.34±0.32	1.07	0.287	−0.04±0.09	−0.45	0.653

## Discussion

Our results suggest that mate-guarding carries no or low energetic costs in male long-tailed macaques because males seem to compensate any reduction in energy intake by lowered energy expenditure. Even though, in our study, males spent less time feeding and fed less on fruits when mate-guarding, UCP measures showed that male energetic status was not affected by mate-guarding. Most likely the reduction in vertical locomotion observed in our study males during mate-guarding counterbalanced reduced food consumption.

Our study confirms previous findings that mate-guarding leads to reduced feeding time in primate males (baboons, *P. cynocephalus*—Rasmussen 1985; Alberts et al. 1996 and *P. anubis*—Packer 1979 and Japanese macaques, *Macaca fuscata*—Matsubara 2003) and males of other taxa (e.g. reptiles, Censky 1995; Ancona et al. 2010 and birds, Komdeur 2001). Males in general thus seem to trade-off time and energy devoted to feeding with that needed for mate-guarding females. The costs they face in terms of reduced feeding time most likely translates into reduced energy intake. This reduction in feeding time may steam from increased time spent on monitoring the guarded female and her environment (i.e. for detection of potential competitors) or from the need to adjust activity to that of the guarded female. In primate species with pronounced sexual size dimorphism, cycling females typically devote less time to feeding activity than males, because of lower energy demands (e.g. chacma baboons, *P. hamadryas ursinus*—Weingrill et al. 2003). Similarly, in a cetacean (the Dall’s porpoise, *Phocoenoides dalli*) males undertake shorter dives when paired with females than when paired with males (Willis and Dill 2007) thus potentially lowering their foraging efficiency. Our study males did not only devote less time to feeding during mate-guarding, they also fed less on fruits, independent of fruit availability, further suggesting decreased energy intake associated with mate-guarding behaviour. Fruits seem to be an important energy source in long-tailed macaques as females time their reproduction with fruit abundance (van Schaik and van Noordwijk 1985). Similarly in whiptail lizards (*Aspidoscelis costata*), males feed less during mate-guarding and also capture smaller prey (Ancona et al. 2010). Future studies should thus consider changes in an individual’s activity budget but also on the animal’s diet when investigating energetic costs of behavioural strategies.

Although in our study, male long-tailed macaques seemed to face a reduction in energy intake during mate-guarding, their overall energetic status (as indicated by our measure of UCP levels) was not affected by this behaviour. The most likely explanation is that, during mate-guarding, reduced energy intake was counterbalanced by a reduction in energy expenditure. In fact, our study males travelled less distance during mate-guarding, though not horizontally as observed in terrestrial baboons (Altmann et al. 1996), but vertically. Climbing might be an important parameter affecting energetic status (our results Table 6, see also Hanna et al. 2008), in contrast to moving on the ground, which seems to represent only a negligible portion of the total energy expenditure (Altmann 1987). A reduction in vertical locomotion may thus be sufficient to counterbalance any decrease in food intake. It remains however unclear from our study whether this reduced climbing occurred as a male compensation strategy or whether it was a side effect of the male adjusting his locomotion to the female.

Interestingly, even though high-ranking male long-tailed macaques mate-guard females more extensively than low-ranking ones (Engelhardt et al. 2006), rank did not affect male energetic status, further indicating that mate-guarding is not energetically costly in this species. By contrast, in rhesus macaques, in which mate-guarding intensity is also rank-dependant, high-ranking males bear a clear cost to this behaviour leading to significantly lower UCP levels at the end of the reproductive season (Higham et al. 2011a). The reason why long-tailed macaques differ from rhesus macaques in this respect may lie in the difference of reproductive seasonality between the two species and in the way male hierarchies are formed. In strictly seasonally breeding species, such as rhesus macaques (Hoffman et al. 2008), males typically store fat prior to the mating season and subsequently face a dramatic degradation of their body condition towards the end of this season (Bernstein et al. 1989; Cooper et al. 2004). Males of non-strictly seasonal primates, such as long-tailed macaques (van Schaik and van Noordwijk 1985), in which females cycle at any time of the year (though with certain peaks) may adopt a different energy management strategy. Males need to maintain a sufficient energy level to respond to the challenges of reproductive competition for much longer (i.e. endurance rivalry, Andersson 1994) and thus cannot afford to completely deplete their energy reserves. Furthermore, whereas male rhesus macaques attain high dominance status through succession or queuing (Berard 1999), high-ranking male long-tailed macaques maintain their rank through contest competition (van Noordwijk and van Schaik 1985). This means that high-ranking long-tailed macaques, in order to maintain their status, need to be prepared for rank challenges year-round whereas rhesus macaques don’t necessarily have to be prepared for those at all. This may even further have selected, in non-strictly seasonal species, for males who are able to balance energy expenditure and intake during mate-guarding so as to never reach a critical point at which their energetic status would be substantially negatively affected.

Another important parameter which may influence the costs of mate-guarding and the ability of males to sustain these costs is the age of the male. In our study, we did not include male age in our analyses since all the high-ranking males were in their prime, and hence of similar age. This pattern is typical for species in which males achieve top dominance rank through contest competition (van Noordwijk and van Schaik 2004). In these species, only males in best bodily condition, thus prime males, can reach alpha-male position. By contrast, in species in which males achieve dominance through queuing, high-ranking males may be of various ages (van Noordwijk and van Schaik 2004) and are more likely to be post-prime. Further studies are however needed to evaluate the effect of age on the costs of mate-guarding in species in which males queue to achieve top dominance rank.

Given that we did not find any energetic costs to mate-guarding, the questions remains why alpha male long-tailed macaques, do not always mate-guard females even when there is only a single one being in her fertile phase (Engelhardt et al. 2006).

One possible explanation may be that alpha males provide reproductive concession to other group males (concession model, Clutton-Brock 1998). The main underlying assumption of the concession model is that the highest ranking individual has complete control over all the reproductive opportunities in his group (Clutton-Brock 1998). This criteria may be met in long-tailed macaques as alpha males are able to discern the females’ fertile phase (Engelhardt et al. 2004) are not energetically limited in their ability to mate-guard females (this study) and female fertile phases do not overlap (Engelhardt et al. 2006). Furthermore, male long-tailed macaques have been observed cooperating with each other to prevent extra group males from accessing females (Girard-Buttoz., unpublished data). Alpha males may thus reproductively benefit from giving concessions to other group males.

Whether the concession model applies to primates is highly debated (Port and Kappeler 2010) and only one study so far claims to provide empirical evidence for its existence. In male chacma baboons, alpha males seem to reduce potential risk of infanticide and enhance their tenure length through reproductive concession (Henzi et al. 2010). A detailed study on male cooperation and the associated fitness benefits for alpha males will be needed to determine whether reproductive concessions indeed takes place in long-tailed macaques or not.

An alternative explanation for why alpha male long-tailed macaques do not mate-guard females as extensively as they theoretically could is that they are limited by other costs than energetic ones. For instance mate-guarding has been shown to be associated with increased aggression rate and/or stress levels in a variety of taxa (baboons, Bercovitch 1983; Bergman et al. 2005; lizards, Ancona et al. 2010; and octopus, Huffard et al. 2010). Further studies are needed in order to evaluate other potential costs (e.g. stress and aggression) connected with mate-guarding in long-tailed macaques.

Overall, our results show that mate-guarding may not be energetically costly across all primate species and suggest that under certain circumstances, males may be able to keep their energy budget balanced even when extensively following a female. Whether our finding can be extrapolated beyond long-tailed macaques or not will need further investigation. There might be, however, species-dependent differences in the degree to which males are able to balance energetic needs, particularly in respect to reproductive seasonality. Our results further illustrate the importance of measuring parameters of both energy intake and expenditure and of controlling for environmental factors such as fruit availability when investigating energetic costs of behavioural strategies. Even more importantly, they show that a combination of behavioural observation with more objective measures of physiological status is necessary for a comprehensive picture of this issue.

More studies using physiological measures are now required to further demonstrate and explain the presence or absence of energetic limitations of mate-guarding. Furthermore, assessing the energetic costs associated with this behaviour is just a first step in understanding the factors constraining male reproductive decisions in multi-male primate groups. An evaluation of other costs (e.g. stress and aggression) is also needed for our understanding of the deviance from the Priority of Access model observed in certain species.
